# DNA methylation orchestrates secondary metabolite biosynthesis and transport in *Papaver somniferum*

**DOI:** 10.1371/journal.pone.0329855

**Published:** 2025-08-25

**Authors:** Tuba Aksoylu, Mine Turktas

**Affiliations:** Department of Biology, Faculty of Science, Gazi University, Ankara, Türkiye; Dokuz Eylul Universitesi, TÜRKIYE

## Abstract

**Background:**

*Papaver somniferum* (opium poppy) is a major source of benzylisoquinoline alkaloids (BIAs), including pharmaceutically important compounds such as morphine and noscapine. While the enzymatic pathways underlying BIA biosynthesis are well-characterized, the epigenetic mechanisms that govern tissue- and genotype-specific alkaloid accumulation remain poorly understood.

**Results:**

This study presents a comparative DNA methylation analysis of stem and capsule tissues from *P. somniferum* cultivars with distinct alkaloid profiles. High-alkaloid-yielding cultivars exhibited hypomethylation of genomic regions involved in photosynthesis, carbon metabolism, protein phosphorylation, and intracellular transport, particularly in stem tissues. DNA methylation patterns revealed tissue- and compound-specific epigenetic signatures: morphine-rich cultivars showed enrichment of differentially methylated regions (DMRs) associated with membrane-related functions, whereas noscapine-rich cultivars exhibited DMR enrichment in nuclear regulatory components and ribosome-associated pathways. Genes encoding cytochrome P450 enzymes, F-box proteins, and ABC transporters were differentially expressed and epigenetically modulated, reflecting a multi-layered regulatory network coordinating biosynthesis, transport, and detoxification of alkaloids.

**Conclusions:**

Our findings suggest that noscapine biosynthesis is under strict, evolutionarily conserved regulatory control, while morphine production is supported by transcriptional and metabolic enhancements in photosynthesis and carbohydrate metabolism. This study provides the first integrative epigenomic perspective on alkaloid biosynthesis in the opium poppy and highlights DNA methylation as a key determinant of metabolic specialization.

## Introduction

Opium poppy (*Papaver somniferum* L.) belongs to the Papaveraceae family, and holds significant medicinal and industrial importance due to its production of benzylisoquinoline alkaloids (BIAs), a structurally diverse group of secondary metabolites. The plant accumulates various pharmacologically active BIAs, including papaverine, codeine, morphine, noscapine, and sanguinarine [1]. More than 2,500 metabolites have been identified as intermediates in the BIA biosynthetic pathway. In opium poppy, over 80 distinct alkaloids have been isolated to date [2]. Six countries are the main legal manufacturers of opium poppy under the supervision of the United Nations Organization.

The biosynthesis and accumulation of BIAs require the involvement of multiple phloem cell types. These processes occur through the transport of metabolites between companion cells, sieve elements, and laticifers. While BIA biosynthesis primarily takes place in stem laticifers, opium alkaloids predominantly accumulate in the capsule [3–7]. The biochemistry and physiology of BIA metabolism in opium poppy have been extensively investigated. Recent studies suggest that the opium poppy genome has an ongoing evolutionary diversification, potentially driven by selective pressures to enhance its functional capacity for medicinal and industrial alkaloid production [7–9].

The biosynthesis of BIAs in opium poppy is a complex pathway involving a diverse array of enzymes, with more than 35 enzymes identified to date as participating in this metabolic pathway [1,10,11]. BIA biosynthesis begins with the condensation of two tyrosine-derived precursors, dopamine and 4-hydroxyphenylacetaldehyde (4-HPAA), catalyzed by the enzyme norcoclaurine synthase (NCS), to produce (S)-norcoclaurine. Subsequently, (S)-norcoclaurine is converted into (S)-reticuline, a central branch-point intermediate. From this point, (S)-reticuline is further metabolized through distinct branches of the pathway to yield various alkaloid end products [1,7,9,12]. In the morphinan branch of the pathway, several key enzymes are involved, including reticuline epimerase (REPI) [13–16]. In the noscapine branch, the enzymes berberine bridge enzyme (BBE), scoulerine 9-O-methyltransferase (SOMT), and noscapine synthase (NCS) play key roles [5].

Epigenetic regulation encompasses three fundamental mechanisms: DNA methylation, histone modifications, and non-coding RNAs, all of which mediate reversible changes [17]. DNA methylation in plants occurs at the cytosine nucleotide, with DNA methyltransferase enzymes (DNMT) playing a crucial role in this process. In plants, DNA methylation is observed at three distinct sequence contexts: CpG, CpHpG, and CpHpH (where H represents A, T, or C). Plants utilize epigenetic mechanisms as an adaptive strategy to regulate gene expression [18,19]. DNA methylation must be precisely regulated, as this regulation is crucial for optimal growth and development in plants [20].

The biosynthesis and accumulation of secondary metabolites in plants vary among species. During the biosynthesis process, gene expression is tightly regulated. One of the primary regulators of plant secondary metabolite biosynthesis is transcription factors. Transcription factors play significant roles in the biosynthesis of BIAs in *P. somniferum* [21]. A large number of studies conducted to elucidate the biosynthetic pathways of secondary metabolites produced by the opium poppy. In this context, numerous transcriptomic studies were carried out using different varieties of opium poppy [12,21–25]. However, only one focused on DNA methylation as an aspect of epigenetic regulation [26]. The results highlighted the influence of DNA methylation on BIA biosynthesis in opium poppy, with cultivars exhibiting distinct BIA profiles showing varying methylation patterns. During BIA biosynthesis, morphine and noscapine are synthesized from S-reticuline through separate pathways, yet the underlying driving force behind this bifurcation remains unclear. In this study, DNA methylation landscapes of three *P. somniferum* L. cultivars were built, and it was aimed to comprehensively analyze organ-specific and cultivar-specific methylation marks to elucidate the factors influencing BIA biosynthesis in opium poppy using next generation sequencing technologies.

## Material method

### Plant material

In this study, three poppy (*Papaver somniferum* L.) cultivars developed and registered by the Turkish Grain Board (TMO) were used as plant material, each characterized by distinct alkaloid profiles. The cultivar Ofis_NP contains a high level of noscapine (1.3%), Ofis_1 is rich in morphine (1.8%), while Ofis_96, with low levels of both morphine (0.6%) and noscapine (0.02%), was used as the control. Plant samples were collected in June from the experimental fields of the Bolvadin District Opium Alkaloids Factory Directorate, located in Afyonkarahisar Province, Turkey.

Plants were grown until seed formation, and capsules and stems (3 cm long) were collected during the flowering period, when alkaloid production is at its peak [27]. Samples were immediately frozen in liquid nitrogen and stored at −80 °C until use.

### DNA isolation and sequencing

Genomic DNA was extracted using the GeneMATRIX Plant&Fungi DNA Purification kit based on the manufacturer’s procedure. Three biological replicates from the stem and capsule organs of each of the three poppy plant varieties were used (two replicates were used for Ofis_NP capsule sample), and the isolated samples were stored at −20 °C until use. High-quality DNA was used for library preparation. Reduced representation bisulfite sequencing (RRBS), an efficient form of bisulfite sequencing (BiSeq) that combines BiSeq with restriction digestion of genomic sequences targeting CpG-rich DNA regions, was applied. Briefly, genomic DNA was cut with the *MspI* restriction enzyme, which cuts CpG-rich regions. Then DNA fragments were treated with sodium bisulfite. Unmethylated Lambda DNA was added to samples to estimate the efficiency of bisulfite conversion and the error rate. Sequencing in a paired-end mode on the Illumina HiSeqX Ten platform generated 150 bp reads.

### Quality control and mapping

Raw reads generated from the Illumina pipeline in FASTQ format were pre-processed to remove low-quality bases and adaptor sequences using TrimGalore v.0.6.10. After quality control with FastQC v.0.11.9, the clean reads were aligned to *P. somniferum* genome (GCF_003573695.1_ASM357369v1) using Bwa-meth v.0.2.2 [28]. Only the reads mapped at the unique position were retained for methylation calling.

### Methylation calling

*R* package MethyKit v.1.29.1 [29] was used to identify differentially methylated regions (DMR) between the samples. The methylation levels in the gene body and flanking regions (2 kb of an upstream or downstream region) were determined by partitioning the sequence into 100 bins of equal size and evaluated as a weighted methylation level.

The DNA methylation level at each mCytosine site was determined by the percentage of reads supporting mC to the total C and T at the same site. Bases that have more than the 99.9^th^ percentile of coverage in each sample were removed, and the true methylated sites were selected with q-value ≤ 0.01, methylation difference ≥ 25%, and sequencing depth ≥ 10. The normalization process was performed to equalize the read coverage in the samples, and the reads of biological replicates were merged.

Correlation coefficients and changes in DNA methylation of CpG, CHG, CHH (where H is any base except G) sequences between the samples were calculated. *R* package with ggplot2 version 3.5.1 was used to create Manhattan plot.

### Gene ontology and functional annotation analysis

GO functional enrichment analysis of genes associated with hyper- and hypomethylated DMRs was carried out using DAVID, and three sub-ontologies were examined as biological process (BS), cellular component (CC), and molecular function (MF) [30,31]. GO terms with corrected q-values less than 0.01 were selected as significantly enriched. Enrichment analysis based on KEGG was performed [32]. Blast analysis was employed to annotate the DMR sequences [33].

## Results

### Sequencing quality

To elucidate the epigenetic contribution of DNA methylation to the variation in benzylisoquinoline alkaloid (BIA) content among different opium poppy (*Papaver somniferum*) cultivars, Reduced Representation Bisulfite Sequencing (RRBS) was performed. The raw sequence data have been deposited in the NCBI database under accession number SRP566503. The bisulfite conversion efficiency was high, with an average conversion rate of approximately 99.3%, indicating reliable detection of methylated cytosines. Sequencing quality was robust, as reflected by consistently high Q20 and Q30 scores (S1 Table). The average GC content of the reads was determined to be 26.7% (S1 Table), consistent with expectations for bisulfite-treated libraries. To determine DNA methylation estimates using bisulfite conversion rates, typically at least 5 × read coverage is required [34]. Approximately 10 Gb of high-quality sequencing data were generated, at an average sequencing depth of approximately 12.3 × per sample (S1 Table, S2 Table). The high-quality filtered reads were uniquely mapped to the *P. somniferum* L. genome, with an average mapping rate of 97.5% (S2 Table).

**Fig 1:**
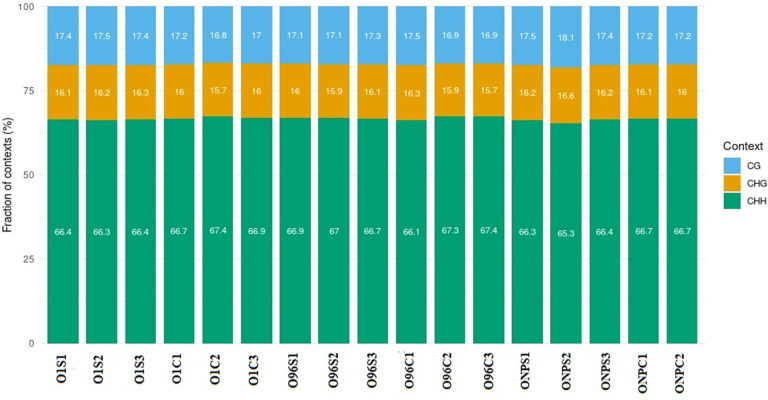
Proportion of CG, CHG, and CHH contexts in the total number of methylation sites.

The 5-mC mark was found in the CG, CHG, and CHH contexts. Average methylation levels of CHG and CHH were %16 and %67, while that of CpG was %17 (Fig 1). The increase in CHH methylation observed in the present study aligns with the findings of a previous investigation conducted in a distinct plant species, suggesting a potentially conserved epigenetic response mechanism across different taxa [35].

**Fig 2:**
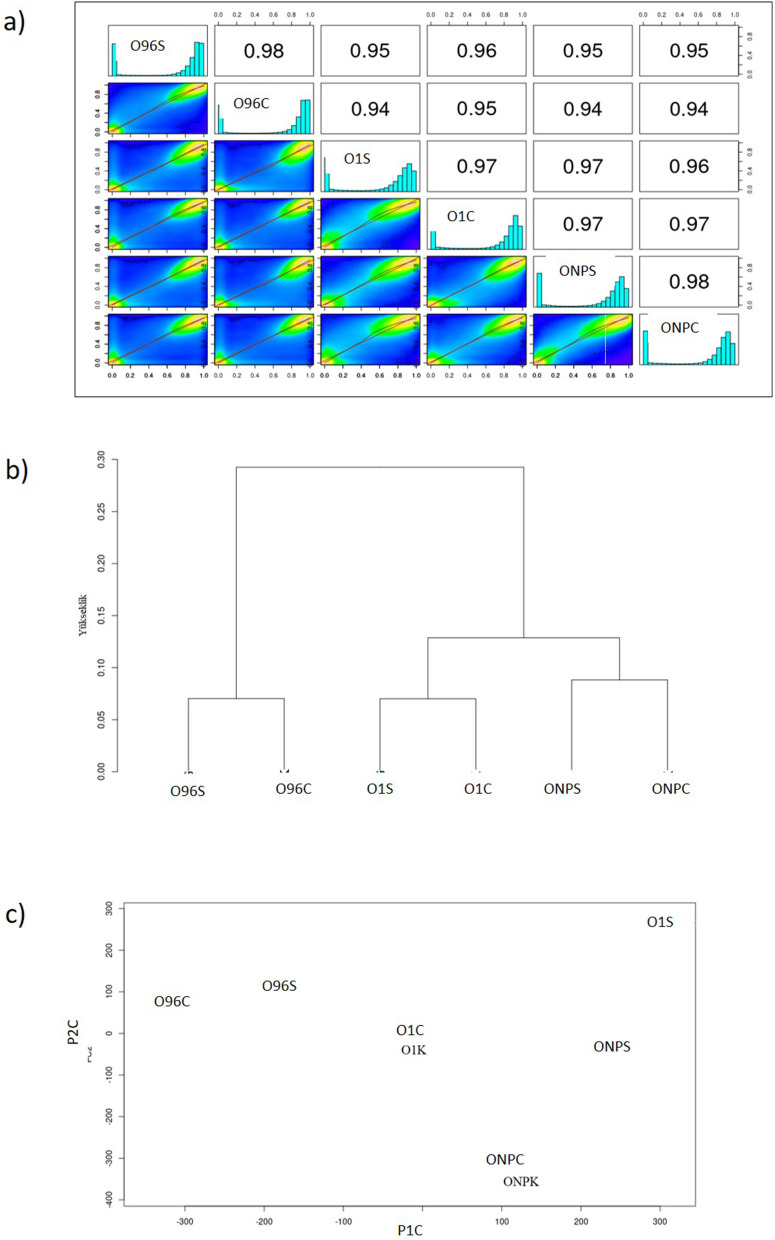
a) Correlation coefficiencies for CpG, b) Cluster analysis for CpG, c) Principal component analysis for CpG.

To assess sample clustering and methylation pattern correlations, Pearson correlation analysis and Principal Component Analysis (PCA) were conducted. CpG methylation profiles exhibited strong positive correlations among the nine comparison groups, with Pearson correlation coefficients ranging from 0.94 to 0.98 (Fig 2a), indicating overall similarity in methylation patterns across samples. Despite this global similarity, hierarchical clustering of samples based on the mean methylation rates suggests a potentially biologically meaningful divergence associated with BIA content (Fig 2b). PCA of CpG methylation landscapes revealed partial separation along the first two principal components (Fig 2c). Notably, the segregation between control group and the alkaloid-rich cultivars highlighted the potential influence of BIA content on DNA methylation dynamics. These findings suggest that genes involved in BIA biosynthesis may be subject to epigenetic regulation. The data for the other two methylation contexts are provided in S1 Fig.

### Analysis of differentially methylated regions

Mapped reads were categorized into nine comparison groups according to cultivar and organ type to facilitate the identification of differentially methylated regions (DMRs) (Table 1).

**Table 1:** List of the comparison groups.

	Compared Sample 1	Compared Sample 2	Comparison Abbreviation
**Cultivar-Based**	Ofis_96_Stem	Ofis_1_Stem	O96S_O1S
	Ofis_96_Stem	Ofis_NP_Stem	O96S_ONPS
	Ofis_NP_Stem	Ofis_1_Stem	ONPS_O1S
	Ofis_96_Capsule	Ofis_1_Capsule	O96C_O1C
	Ofis_96_Capsule	Ofis_NP_Capsule	O96C_ONPC
	Ofis_NP_Capsule	Ofis_1_Capsule	ONPC_O1C
**Organ- Based**	Ofis_96_Capsule	Ofis_96_Stem	O96C_O96S
	Ofis_1_Capsule	Ofis_1_Stem	O1C_O1S
	Ofis_NP_Capsule	Ofis_NP_Stem	ONPC_ONPS

**Fig 3:**
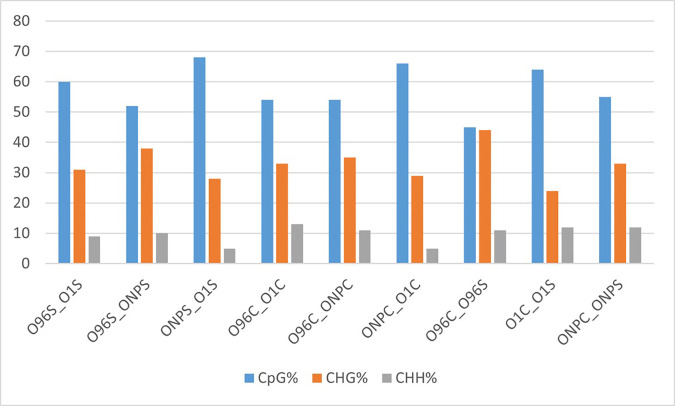
Percentage methylation levels of DMRs identified for each comparison group across CpG, CHG, and CHH contexts.

DNA methylation levels of differentially methylated regions were profiled across all three sequence contexts (CpG, CHG, and CHH) for each comparison group. Among these, CpG context showed the highest level of DNA methylation, followed by CHG and CHH contexts (Fig 3).

**Fig 4:**
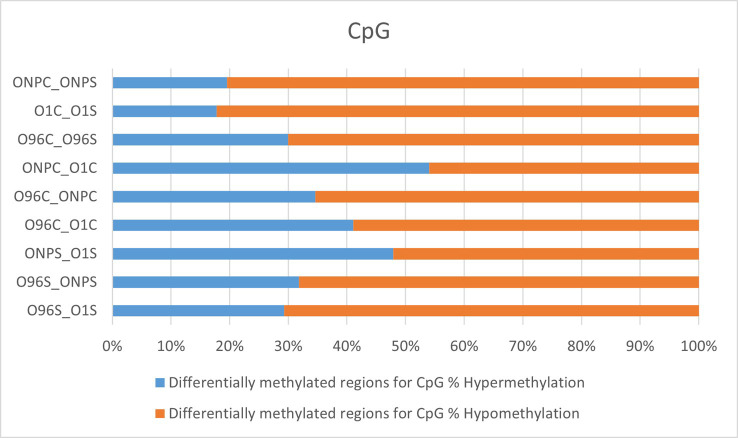
Percentage distribution of hypermethylation and hypomethylation in CpG context among the DMRs identified in the comparison groups.

Hypermethylated and hypomethylated CpG sites were identified across all comparison groups, and it was found that the rate of hypomethylation is higher than that of hypermethylation (Fig 4). Relative to the control cultivar, Ofis_1 and Ofis_NP – both characterized by high BIA content-exhibited a greater number of hypomethylated CpG sites, with this trend being particularly pronounced in stem tissues. In contrast, a more balanced distribution of hyper- and hypomethylated CpG sites was observed in the comparison between these two high-BIA cultivars. The data for the other two methylation contexts are provided in S2 Fig.

**Fig 5:**
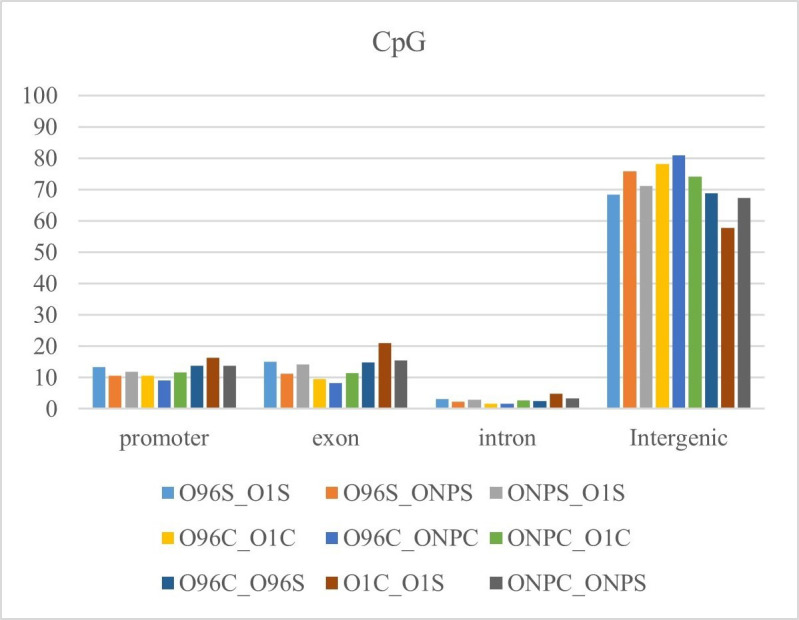
Genomic distribution of methylation differences of CpG context.

Analysis of the genomic distribution of methylation differences showed that they predominantly occur in intergenic regions (Fig 5). The data for the other two methylation contexts are provided in S3 Fig.

**Fig 6:**
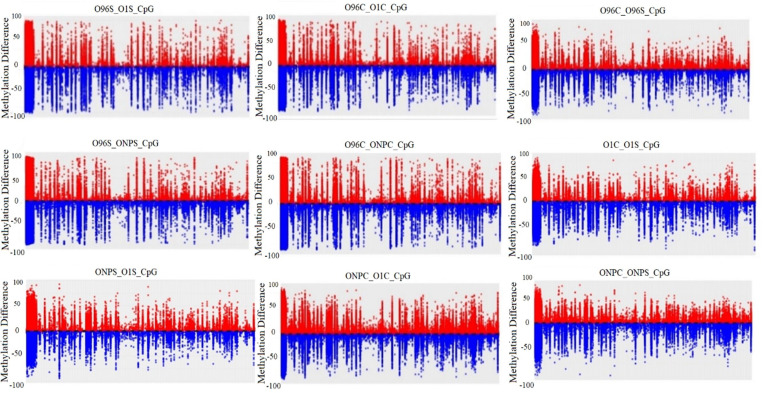
Distribution of hyper- and hypomethylation in terms of CpG methylation based on cultivar and organs according to Manhattan plot graph (Red: Hypermethylation Blue: Hypomethylation).

The Manhattan plot illustrates the distribution of CpG methylation levels across the comparison groups (Fig 6). While methylation levels were predominantly concentrated around 25%, the majority of DNA regions exhibited low levels of methylation. The data for the other two methylation contexts are provided in S4 Fig.

### Functional enrichment of DMR-associated genes

Gene Ontology (GO) enrichment and pathway analyses were performed to explore the functional significance of genes associated with DMRs across all comparison groups. GO annotations were categorized under three main domains: Biological Process (BP), Molecular Function (MF), and Cellular Component (CC). Enrichment was assessed using DAVID with a q-value threshold of <0.01 for statistical significance. Redundant GO terms were filtered, and the most representative terms were visualized (S5 Fig). Organ-specific and cultivar-specific differences in GO term distribution were examined to identify biological processes and cellular structures potentially regulated via DNA methylation (S1 File). Differential methylation analysis revealed variations in DNA methylation across nine comparison groups.

When analyzed, “*protein phosphorylation*” and “*transmembrane transport*” were identified as the most frequently enriched Biological Process (BP) terms for both organ types and cultivars. Within the Cellular Component (CC) category, “*nucleus*” was the most commonly represented term overall. However, membrane-related terms were particularly enriched in the differentially methylated regions between the stems of the two BIA-rich cultivars. A detailed analysis of CC GO terms associated with differentially methylated regions (DMRs) revealed that the “*nucleus*” was the most enriched term among hypomethylated DMRs in the BIA-rich cultivars compared to the control sample. In the GO analysis of hypermethylated DMRs identified from the comparative analysis of stem tissues of two BIA-rich cultivars, distinct patterns of subcellular localization were observed. In the morphine-rich Ofis_1 cultivar compared to noscapine-rich Ofis_NP cultivar, hypomethylated DMRs were most significantly enriched in the “*plasma membrane*” GO term, whereas hypermethylation was predominantly associated with regions linked to the “*extracellular region*”. These findings suggest cultivar-specific epigenetic modifications that may influence cellular compartmentalization relevant to alkaloid biosynthesis. Besides, in the organ-based comparison, Ofis_NP and Ofis_96 exhibited similar profiles, with “*nucleus*” emerging as the most prominent CC term. In contrast, Ofis_1 organs exhibited a distinct profile. Specifically, the term “*plasma membrane*” was associated with hypomethylated DMRs in the stem, while the term “*cytoplasm*” was enriched in the capsule.

Interestingly, among the comparison of stems of the cultivars, the term “*regulation of DNA-templated transcription*” emerged as one of the most enriched biological process (BP) terms associated with hypomethylated differentially methylated regions (DMRs) was found specifically in Ofis_1 compared to Ofis_96. In the Molecular Function (MF) category, “*protein binding*” and “*ATP binding*” were consistently among the most represented terms across all comparison groups.

In the stem of all the cultivars, the most enriched BP terms associated with hypomethylated DMRs, relative to capsules, were “*phosphorylation*”, “*transmembrane transport*”, and “*proteolysis*”. However, a particularly noteworthy observation only in the Ofis_1 variety was the significant enrichment of the “*carbohydrate metabolic process*” BP term-closely associated with photosynthetic activity-among the most frequently identified hypomethylated DMR-related categories. This was further supported by the overrepresentation of the KEGG pathway “*starch and sucrose metabolism*” among hypomethylated regions, reinforcing the potential role of energy metabolism in Ofis_1 stem tissues. Given the substantial energy demands of alkaloid biosynthesis, these findings suggest that photosynthetically derived carbohydrate pathways may serve as a primary metabolic source to support this process. The pronounced hypomethylation in photosynthesis-related terms, particularly in Ofis_1, may reflect an epigenetically regulated enhancement of photosynthetic efficiency, potentially contributing to higher morphine biosynthesis in this cultivar. Together, these results highlight a cultivar-specific epigenetic modulation of key metabolic pathways, in line with the broader aim of this study to uncover the epigenetic basis of physiological and biochemical diversity among *Papaver somniferum* cultivars.

Pathway enrichment analysis revealed that the impact of BIA production in stem tissues on overall DNA methylation was primarily associated with pathways related to “*biosynthesis of secondary metabolites*” and “*biosynthesis of cofactors*”. In contrast, the diversity of BIA compounds appeared to most strongly influence the “*endocytosis*” pathway. Furthermore, in the morphine-rich cultivar compared to noscapine-rich cultivar, genes associated with the “*endocytosis*” pathway were found to be hypermethylated in stem tissues which suggests a potential epigenetic suppression of endocytosis-related genes in response to elevated morphine biosynthesis. In support of the inhibition along the endocytic pathway, the “*Golgi apparatus*” CC term is notably enriched among the hypermethylated differentially methylated regions (DMRs) in the capsule tissue of the morphine-rich Ofis_1 variety. This enrichment suggests a potential epigenetic modulation of Golgi-related processes, which may be linked to altered cellular trafficking and processing pathways.

Compared to the control, “*biosynthesis of cofactors*”, “*ubiquitin-mediated proteolysis”* and *“mRNA surveillance pathway*” were the most enriched KEGG pathways identified exclusively for the hypomethylated DMRs of noscapine-rich cultivar compared with control in the stems. The result highlights the involvement of post-translational modification in noscapine-rich cultivar. This enrichment may reflect differences in mRNA surveillance activity in the noscapine-rich variety, potentially indicating a distinct genetic or epigenetic landscape that influences noscapine biosynthesis. Among capsule tissues of different cultivars, “*plant hormone signal transduction*” emerged as the most significantly enriched KEGG pathway.

The pathway analysis of hypomethylated DMRs in the capsules of Ofis_1 and Ofis_NP, compared to the control, revealed that the most enriched pathway was “*ATP-dependent chromatin remodeling*”. However, this pattern changed when comparing alkaloid-rich samples, where the “*biosynthesis of secondary metabolites*” pathway was found to be hypomethylated in Ofis_1 capsules relative to Ofis_NP. Another remarkable finding is that, “*metabolic pathways*” was significantly enriched for hypomethylated regions obtained from Ofis_NP capsule compared to Ofis_1 capsule. Previous studies have demonstrated that the biosynthesis of BIAs occurs not only in stem tissues but also in the capsule organ [36]. The findings of the present study further support this observation. Notably, the distinct epigenetic regulatory profile observed in the noscapine_ cultivar, compared to morfin-rich cultivar, may also indicate the existence of uncharacterized intermediate routes involved in noscapine biosynthesis – an area that has also been highlighted as not yet fully elucidated in previous studies [37].

In the organ-based comparison, the most abundand CC term was found to be “*nucleus*” for hypomethylated DMRs in stem of both Ofis_96 and Ofis_NP cultivars compared to capsule. In contrast, “*plasma membrane*” was identified as the predominant CC term for stem of Ofis_1.

### Functional enrichment of DMR-associated genes in promoter regions

It is well-established that DNA methylation in promoter regions regulates gene expression and this epigenetic modification serves as a crucial mechanism for the regulation of various biological processes [38]. In this context, analyses at the genomic regions level were further elaborated, with a particular focus on promoter regions, where a Gene Ontology (GO) enrichment analysis was performed (S2 File).

In the stem tissues of alkaloid-rich varieties, compared to the control group, the “*protein phosphorylation*” (BP) and “*plasma membrane*” (CC) were found to be the most enriched terms in the promoter regions, mirroring the genomic-wide patterns observed. These terms indicated the pivotal roles of signaling and membrane-associated processes in alkaloid synthesis. Additionally, in comparison to Ofis_NP, the term “extracellular region” and “plasma membrane” were enriched in hypermethylated DMRs in Ofis_1, which may point to changes in the extracellular matrix and signaling pathways that could contribute to the differences in metabolic activity between the two varieties. Another remarkable finding was the significant enrichment of the terms related to “*photosynthesis, light harvesting in photosystem I*” in the hypomethylated DMRs of stems compared to capsules of all cultivars which corroborates the expected active photosynthetic process in the stem tissues. This finding aligns with the physiological need for energy in biosynthesis, particularly for alkaloid production.

Among the CC terms, “*photosystem II*”, “*photosystem I*”, “*chloroplast thylakoid membrane*” and “*plastoglobule*” appeared as the most represented terms in Ofis_1 compared the control group. These terms highlight the involvement of chloroplast-associated structures in the energetic demands of the plant, emphasizing the role of photosynthesis in sustaining high metabolic activity, particularly in morphine-rich cultivars.

Furthermore, the KEGG pathway analysis revealed a significant enrichment of the “*Ribosome*” term in the stem tissues of Ofis_NP compared to Ofis_96, suggesting an enhanced translational regulation process in this variety. This could indicate a higher rate of protein synthesis and potentially reflect a more active cellular machinery in non-alkaloid-producing varieties. These detailed findings not only provide insights into the differential regulation of metabolic processes but also offer a deeper understanding of the epigenetic mechanisms driving alkaloid biosynthesis, cellular transport, and energy production in *P. somniferum* varieties.

### Annotation analysis

In this study, BLAST analysis was performed to annotate DMRs identified for each comparison group. The obtained data are presented in S3 File.

### Annotation of organ-based DMRs

In all three cultivars, gene expression levels were observed to be higher in stem tissues compared to capsule tissues. From this perspective, expression levels of genes such as *ABC transporters***,** *F-box proteins***,** **cytochrome P450s, and* *pentatricopeptide repeat-containing** proteins were found to be upregulated by up to threefold in the stem tissues of all cultivars. These gene families, while functionally diverse, are commonly associated with secondary metabolism, stress responses, and cellular regulation. Their coordinated upregulation in stem tissues suggests an integrated role in supporting active biosynthesis, transport, and regulation of specialized metabolites such as BIAs. Given that biosynthesis is expected to occur predominantly in stem tissues, the results of the analysis support this expectation. Additionally, while the *WRKY transcription factor* showed approximately a 20-fold increase in expression in the stem tissues of Ofis_96 and Ofis_1 cultivars, while no significant upregulation was observed in Ofis_NP, which is particularly noteworthy. The analyses were further refined by focusing on regions with higher levels of methylation (≥50%) (S4 File), revealing a similar pattern of gene family upregulation. These findings indicate that these gene families are subject to substantial epigenetic regulation in an organ-specific manner.

### Annotation of cultivar-based DMRs

The expression levels of “*ABC transporter”****, “****purine permease”,* ***“****zinc finger protein”,* and “*F-box protein”* genes were found to be upregulated in both metabolite-rich cultivars compared to the control group. The analysis also highlighted notable changes in the expression of genes involved in the photosynthesis process. Genes such as “*Photosystem II”****, “****sugar transport proteins”,* ***“****pentatricopeptide repeat-containing protein”s*, and “*cytochrome P450s”* exhibited greater expression increases in the metabolite-rich cultivars than in the control. Additionally, the expression of “*chlorophyll a-b binding protein of LHCII”* increased in Ofis_1 and Ofis_NP cultivars relative to the control, although a notable repression was observed specifically in the stem tissues of the Ofis_NP cultivar.

The analyses were further conducted in greater detail at the cultivar level by focusing on regions with methylation levels exceeding 50% (S4 File). *WRKY and MYB transcription factors* tended to show higher expression levels in the Ofis_1 and Ofis_NP cultivars compared to the control group. Additionally*, “F-box proteins”* and “*cytochrome P450s”* showed increased expression particularly in the stem tissues of the metabolite-rich cultivars compared to the control group. “*Pentatricopeptide repeat-containing proteins”* also exhibited higher expression specifically in the Ofis_NP cultivar relative to the control. These findings support the expected high levels of biosynthesis in metabolite-rich cultivars. Moreover, the data confirm that gene distribution at high methylation levels is generally lower in these cultivars, reinforcing the cultivar-specific epigenetic landscape

In connection with the DMRs located in promoter regions, annotation analyses were further refined to identify associated gene expression patterns (S5 File). Compared to the control group, alkaloid-rich varieties exhibited a notable increase in the expression levels of “*F-box protein”* genes, suggesting their potential role in secondary metabolic regulation. When comparing the two metabolite-rich cultivars, Ofis_1 and Ofis_NP, a marked upregulation of “*F-box protein”*, “*Pentatricopeptide repeat-containing protein*”, and “*cytochrome P450”* genes was detected specifically in the stem tissues of Ofis_1. These genes are well-known for their involvement in post-translational regulation, RNA processing, and enzymatic modifications central to secondary metabolism. The elevated expression of these genes in Ofis_1 highlights a distinct transcriptional landscape, pointing to dynamic changes within the secondary metabolite biosynthetic pathways. These findings are consistent with the enhanced biosynthetic activity observed in this variety and further substantiate the epigenetic and transcriptional coordination underlying high alkaloid production.

### Analysis of well-characterized genes functioning in the BIA pathway

The key enzymes involved in the BIA biosynthesis pathway in *P. somniferum* have been previously identified in earlier studies. In our study, among these enzymes, “*tyrosine/DOPA decarboxylase”* and “*S-norcoclaurine synthase”* were observed to be more highly expressed in the Ofis_1 and Ofis_NP cultivars compared to the control group. Additionally, for enzymes involved in the morphine biosynthetic pathway, increased expression levels of “*codeine O-demethylase*” and “*salutaridinol 7-O-acetyltransferase*” were detected in the Ofis_1 and Ofis_NP cultivars relative to the control (S3 File).

## Discussion

The opium poppy (*Papaver somniferum*) is a pharmaceutically valuable species owing to its unique capacity to biosynthesize a wide array of benzylisoquinoline alkaloids (BIAs), which exhibit substantial structural and functional diversity [1]. While extensive research has elucidated the enzymatic and cellular underpinnings of alkaloid biosynthesis and storage [1,3,5–7,12,39], the epigenetic regulatory frameworks that modulate these processes remain largely uncharacterized.

Among various epigenetic mechanisms, DNA methylation is a particularly potent modulator of gene expression. In plants, it operates within a sophisticated network that integrates signal transduction, post-translational modifications, and environmental cues. Within this regulatory landscape, protein phosphorylation emerges as a pivotal process, orchestrating diverse functions including stress responses, hormone signaling, and secondary metabolism [40]. In this context, our comparative analyses revealed pronounced expression of DMRs involved in protein phosphorylation pathways in high-alkaloid-yielding cultivars, particularly in the stem. This suggests a coordinated epigenetic and signaling network that fine-tunes BIA biosynthesis in a tissue- and cultivar-specific manner. The enrichment of phosphorylation-related DMRs especially in the stem tissues of alkaloid-rich varieties underscores a likely post-translational regulatory layer that complements transcriptional control to enable dynamic metabolic reprogramming.

Alkaloid accumulation patterns displayed clear spatial specificity, which is reflected in the transcriptional landscapes of stem and capsule tissues. These patterns are consistent with previous findings that demarcate vascular tissues as the primary sites of alkaloid biosynthesis, whereas capsules function as principal storage organs [6]. The observed compartmentalization of biosynthesis and storage appears to be orchestrated by integrated transcriptional and epigenetic regulation. Notably, inter-cultivar variability in gene expression suggests that epigenetic modulation operates in a genotype-dependent manner to govern organ-specific metabolic activities.

Strikingly, plasma membrane–related processes were particularly enriched in morphine-rich cultivars, as evidenced by the overrepresentation of DMRs linked to membrane components in stem tissues. This highlights the potential involvement of transmembrane signaling and metabolite trafficking in morphine biosynthesis. Conversely, the noscapine-rich cultivar exhibited a DMR enrichment profile associated with nuclear functions, indicative of transcription-centric regulatory architecture. These contrasting patterns suggest that distinct epigenetic strategies have evolved to accommodate the specific biosynthetic demands of different alkaloid types.

Furthermore, the regulation of membrane-associated gene expression via DNA methylation in morphine-rich cultivars suggests a finely tuned mechanism that modulates transmembrane signaling and metabolite transport in response to developmental or environmental stimuli. Such regulation likely facilitates the synchronized expression of biosynthetic enzymes, transporters, and signaling components required for high-yield alkaloid production.

Photosynthesis represents another crucial determinant of secondary metabolite biosynthesis, serving as the principal energy source for metabolically demanding processes [37,41]. The modulation of photosynthetic gene expression, particularly that of photosystem II components, reveals a divergent regulatory strategy between morphine- and noscapine-dominant cultivars. While photosynthesis-related genes were broadly upregulated in the stem tissues of high-alkaloid cultivars, only the morphine-rich cultivar exhibited enhanced expression of photosystem II genes. This suggests a potential epigenetic mechanism that integrates photosynthetic activity with alkaloid biosynthetic pathways in a compound-specific manner.

Photosynthetically derived carbon, primarily in the form of sucrose and starch, also plays a fundamental role in supporting secondary metabolism. Consistent with previous studies showing elevated carbon metabolism under biotic stress [42], we detected hypomethylated DMRs involved in carbohydrate metabolic processes in morphine-rich cultivars. These findings support the hypothesis that both energy production and carbon partitioning are subject to epigenetic control during alkaloid biosynthesis.

Cytochrome P450 (CYP) enzymes serve as a key intersection between primary and specialized metabolism. These enzymes are involved not only in the photoprotective functions of photosynthesis but also in pivotal steps of BIA biosynthesis, such as the stereochemical inversion of (S)-reticuline to (R)-reticuline [7,43]. The enhanced expression of CYP genes in high-metabolite cultivars reinforces their dual functional role. Moreover, the hypomethylation of photosynthesis-associated genes in Ofis_1 may reflect a regulatory mechanism that prioritizes photosynthetic efficiency to support morphine biosynthesis.

Evidence of translational-level regulation also emerged from our analysis. F-box proteins, which mediate selective proteolysis through the ubiquitin–proteasome system, were upregulated in high-alkaloid cultivars [44,45]. Enrichment of genes involved in “ubiquitin-mediated proteolysis” pathways in the noscapine-rich cultivar indicates an additional regulatory tier controlling alkaloid metabolism. Ribosome-associated regulation, including mRNA surveillance and metabolite-sensitive translational stalling [46,47], further underscores the complexity of post-transcriptional control mechanisms. The noscapine-rich cultivar’s enrichment of “*ribosome*” and “*mRNA surveillance*” terms suggests translational precision as a key regulatory feature, in line with prior proteomic studies [37].

Collectively, these findings indicate that noscapine biosynthesis is governed by a highly structured, multi-layered regulatory regime. Unlike morphine biosynthesis-which is hypothesized to have emerged relatively recently in the *Papaver* genus [48] – the noscapine biosynthetic pathway appears to be regulated by evolutionarily conserved mechanisms [49]. The presence of such deeply rooted regulatory elements suggests that noscapine production may be under stricter evolutionary constraints.

In parallel, we identified epigenetic modifications associated with intracellular trafficking systems, including endocytosis and Golgi-mediated vesicular transport. Endocytosis has been implicated in the uptake of external stimuli and the regulation of cellular homeostasis, processes that may indirectly influence alkaloid compartmentalization [50,51]. The DMR enrichment in genes related to endocytosis in both morphine- and noscapine-rich cultivars suggests that vesicle trafficking is epigenetically modulated to support metabolite distribution and detoxification. In the case of morphine-an alkaloid with high cytotoxic potential-limiting endocytic recycling may represent a protective strategy to confine the compound within storage tissues.

The Golgi apparatus, essential for protein processing and vesicle trafficking, also appears to contribute to alkaloid transport [52,53]. Our results suggest that Golgi-related gene expression is altered in Ofis_1, potentially enabling more efficient trafficking and accumulation of morphine in capsules. These observations align with previous findings that secondary metabolites can affect vesicle fusion and membrane dynamics [54], reinforcing the role of epigenetic regulation in shaping transport pathways according to specific metabolic profiles.

Enhanced expression of genes involved in “transmembrane transport” pathways-particularly ATP-binding cassette (ABC) transporters and BIA uptake permeases (BUPs) [7,55–59] – further indicates a robust transcriptional response aimed at optimizing intracellular alkaloid distribution. The upregulation of these transporters suggests that transport systems are reprogrammed in high-yield cultivars to support increased biosynthetic flux while minimizing cytotoxicity.

Finally, the genomic colocalization of GO terms related to biosynthesis, transport, and signal transduction offers additional insight into the coordinated epigenetic regulation of metabolite-related loci. These spatial clusters may function as regulatory hotspots, modulating gene expression in response to environmental and developmental stimuli, and thereby facilitating fine-tuned control of BIA biosynthesis.

In conclusion, this study presents the first comparative DNA methylation analysis of stem and capsule tissues in *P. somniferum* cultivars with contrasting alkaloid profiles. Our findings reveal that the biosynthesis of noscapine is subject to more stringent, multi-tiered regulatory control, whereas morphine production is supported by enhanced photosynthetic and carbon metabolic activity. Together, these insights underscore the critical role of epigenetic regulation in driving the metabolic specialization and diversification of secondary metabolism in the opium poppy.

## Supporting information

S1 Fig a) Correlation coefficiencies for CHG, b) Cluster analysis for CHG, c) Principal component analysis for CHG, d) Correlation coefficiencies for CHH, e) Cluster analysis for CHH, f) Principal component analysis for CHH. (JPG)

S2 Fig a) Percentage distribution of hypermethylation and hypomethylation in CHG context among the DMRs identified in the comparison groups, b) Percentage distribution of hypermethylation and hypomethylation in CHH context among the DMRs identified in the comparison groups. (JPG)

S3 Fig a) Genomic distribution of methylation differences of CHG context, b) Genomic distribution of methylation differences of CHH context. (JPG)

S4 Fig a) Distribution of hyper- and hypomethylation in terms of CHG methylation based on cultivar and organs according to Manhattan plot graph (Red: Hypermethylation Blue: Hypomethylation), b) Distribution of hyper- and hypomethylation in terms of CHH methylation based on cultivar and organs according to Manhattan plot graph (Red: Hypermethylation Blue: Hypomethylation). (JPG)

S5 Fig Top 10 enriched Biological Process (BP), Cellular Component (CC), Molecular Function (MF) terms for all comparison groups. (JPG)

S1 Table Features of the reads. (DOCX)

S2 Table Alignment efficiency of the samples. (DOCX)

S1 File The most represented gene ontology terms in all comparison groups. (XLSX)

S2 File The most represented gene ontology terms in promoter regions of all comparison groups. (XLSX)

S3 File BLAST analysis results of DMRs for each comparison group. (XLSX)

S4 File BLAST analysis results of DMRs for regions with methylation levels exceeding 50%. (XLSX)

S5 File BLAST analysis results of DMRs in the promoter regions of stem organs. (XLSX)
