# Supplementary figures and images for: DNA methylation orchestrates secondary metabolite biosynthesis and transport in Papaver somniferum
Source: PLoS One. 2025 Aug 25;20(8):e0329855. doi: 10.1371/journal.pone.0329855 (PMC12377628; doi:10.1371/journal.pone.0329855)

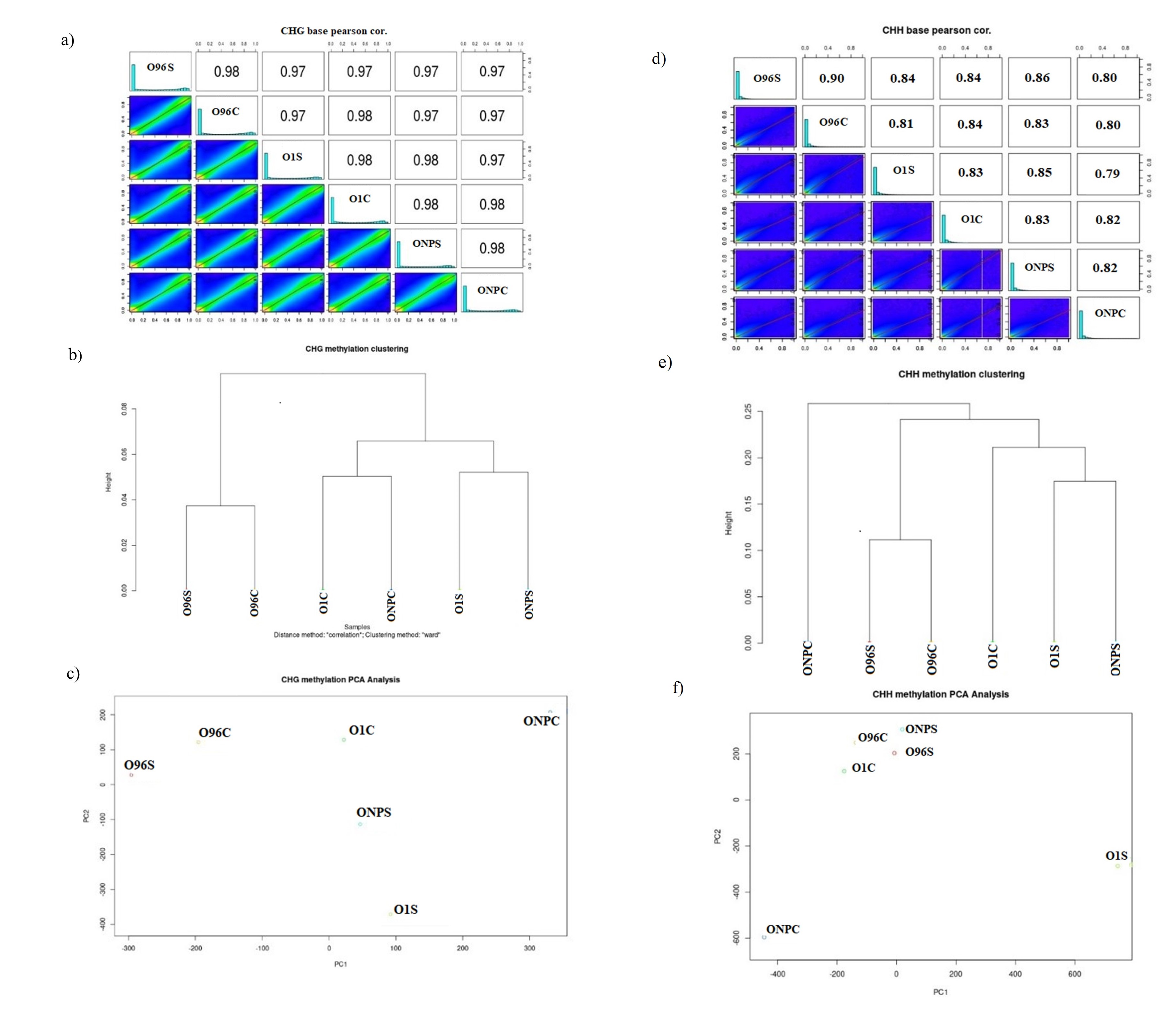

Supplement: S1 Fig — (JPG) [file pone.0329855.s001.jpg]

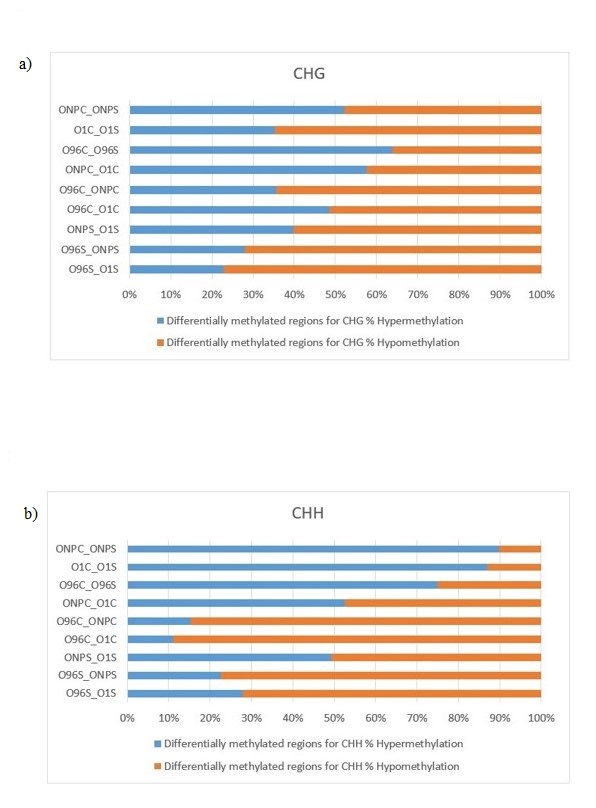

Supplement: S2 Fig — (JPG) [file pone.0329855.s002.jpg]

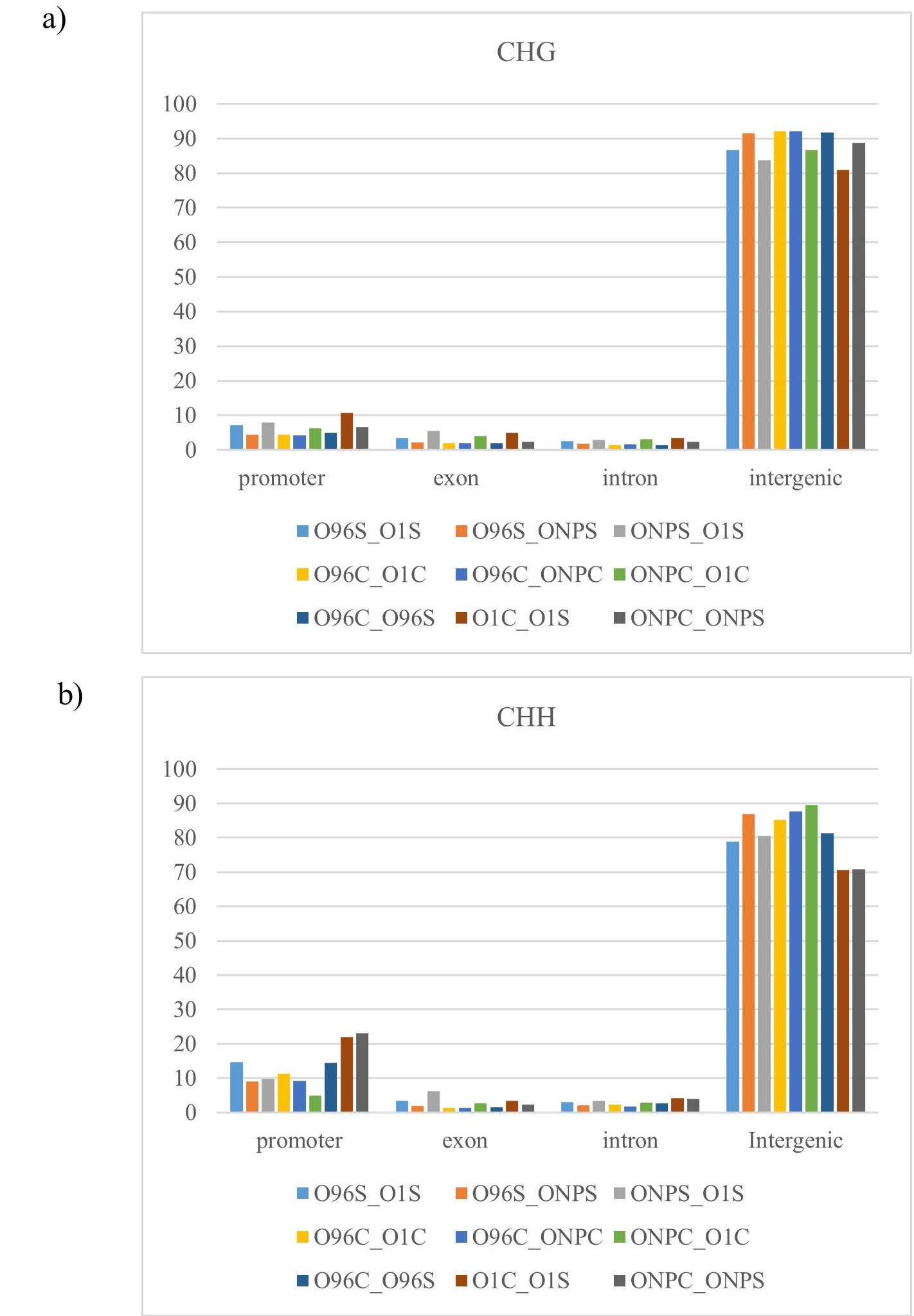

Supplement: S3 Fig — (JPG) [file pone.0329855.s003.jpg]

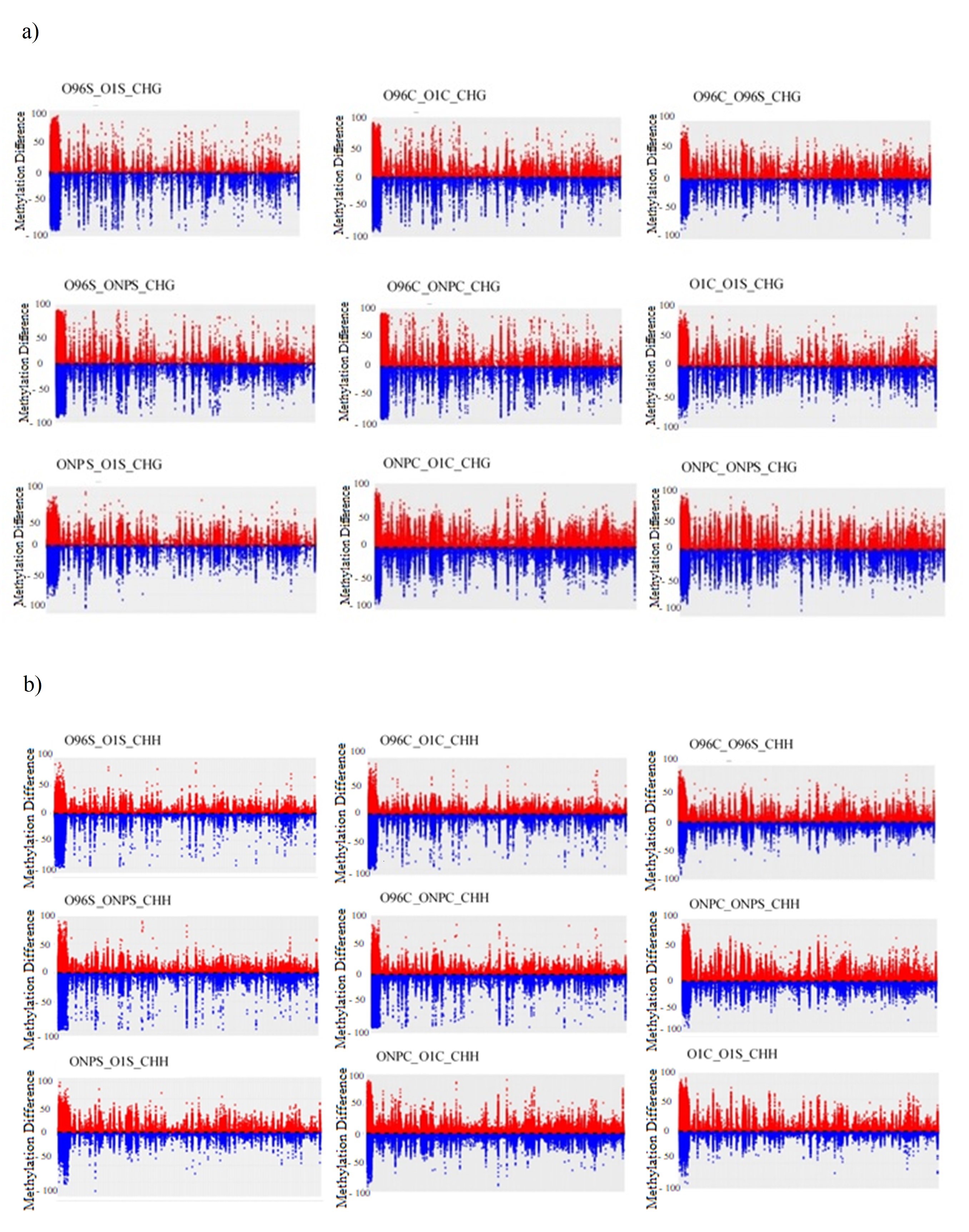

Supplement: S4 Fig — (JPG) [file pone.0329855.s004.jpg]

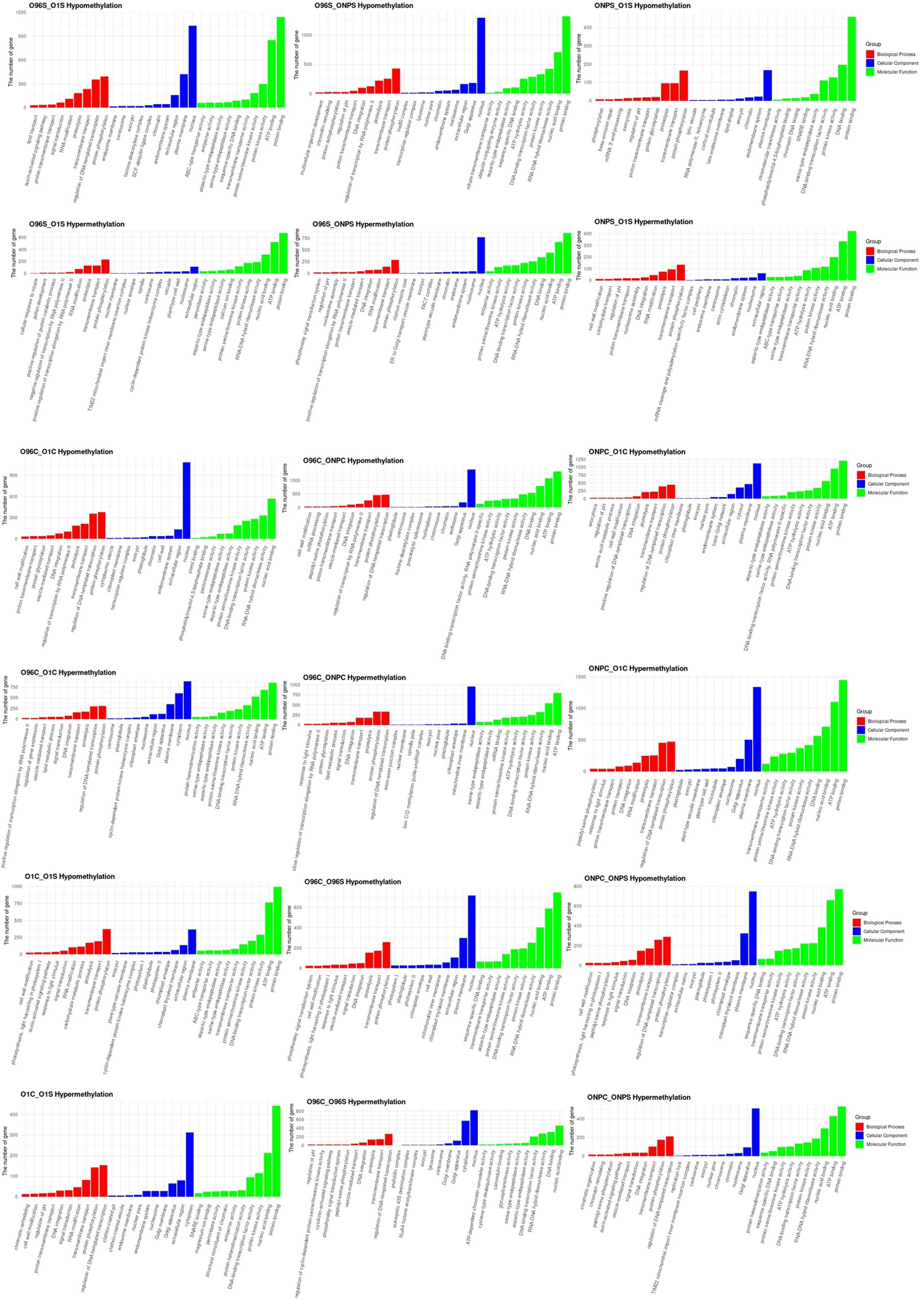

Supplement: S5 Fig — (JPG) [file pone.0329855.s005.jpg]
