# Supplementary material for: DNA methylation orchestrates secondary metabolite biosynthesis and transport in Papaver somniferum
Source: PLoS One. 2025 Aug 25;20(8):e0329855. doi: 10.1371/journal.pone.0329855 (PMC12377628; doi:10.1371/journal.pone.0329855)
Supplement: S1 Table — (DOCX) [file pone.0329855.s006.docx]

Supplementary Table 1. Features of the reads

| **Samples** | ***Msp*I digestion efficiency (%)** | **Number of Methylated C Base** | **Number of Unmethylated C Base** | **Bisulfite conversion rate (%)** | **Total Bases** | **Q20** | **Q20(%)** | **Q30** | **Q30(%)** | **GC(%)** |
| --- | --- | --- | --- | --- | --- | --- | --- | --- | --- | --- |
| Ofis1_Stem_1 | 99.3 | 209,856 | 31,930,796 | 99.3 | 11,464,608,300 | 11,105,592,520 | 96.9 | 10,504,134,275 | 91.6 | 27.5 |
| Ofis1_Stem_2 | 99.3 | 134,132 | 20,023,362 | 99.3 | 10,249,968,000 | 9,893,371,565 | 96.5 | 9,343,120,610 | 91.2 | 29.2 |
| Ofis1_Stem_3 | 99.0 | 219,838 | 37,033,301 | 99.4 | 12,676,343,700 | 12,250,769,975 | 96.6 | 11,590,583,520 | 91.4 | 29.8 |
| Ofis1_Capsule_1 | 99.4 | 78,033 | 13,454,435 | 99.4 | 11,287,218,300 | 10,983,626,148 | 97.3 | 10,409,160,899 | 92.2 | 25.1 |
| Ofis1_Capsule_2 | 98.8 | 106,552 | 18,577,689 | 99.4 | 10,523,686,800 | 10,246,312,416 | 97.4 | 9,725,727,302 | 92.4 | 25.2 |
| Ofis1_Capsule_3 | 99.1 | 93,327 | 15,965,917 | 99.4 | 11,488,569,900 | 11,174,711,848 | 97.3 | 10,584,269,358 | 92.1 | 24.7 |
| Ofis96_Stem_1 | 98.6 | 111,285 | 20,317,012 | 99.5 | 12,584,126,100 | 12,215,505,699 | 97.1 | 11,533,910,541 | 91.7 | 26.8 |
| Ofis96_Stem_2 | 99.5 | 218,079 | 34,470,895 | 99.4 | 11,541,414,300 | 11,230,010,797 | 97.3 | 10,652,286,391 | 92.3 | 25.4 |
| Ofis96_Stem_3 | 99.2 | 90,705 | 15,261,889 | 99.4 | 11,236,037,400 | 10,938,858,511 | 97.4 | 10,367,489,276 | 92.3 | 25.8 |
| Ofis96_Capsule_1 | 97.7 | 228,972 | 41,147,900 | 99.4 | 10,410,751,800 | 10,101,823,660 | 97.0 | 9,545,259,953 | 91.7 | 28.3 |
| Ofis96_Capsule_2 | 98.4 | 208,792 | 38,854,057 | 99.5 | 11,834,760,900 | 11,504,327,394 | 97.2 | 10,895,659,913 | 92.1 | 26.8 |
| Ofis96_Capsule_3 | 98.4 | 201,755 | 31,646,368 | 99.4 | 10,297,960,500 | 10,018,524,663 | 97.3 | 9,503,076,646 | 92.3 | 26.4 |
| OfisNP_Stem_1 | 99.5 | 206,643 | 34,924,548 | 99.4 | 10,228,436,400 | 9,937,150,831 | 97.2 | 9,412,801,137 | 92.0 | 26.6 |
| OfisNP_Stem_2 | 99.3 | 312,786 | 52,442,708 | 99.4 | 10,467,846,300 | 10,173,240,361 | 97.2 | 9,661,893,121 | 92.3 | 28.6 |
| OfisNP_Stem_3 | 99.4 | 220,370 | 35,404,625 | 99.4 | 10,657,930,800 | 10,365,146,451 | 97.3 | 9,837,903,587 | 92.3 | 26.6 |
| OfisNP_Capsule_1 | 99.3 | 54,778 | 9,089,773 | 99.4 | 10,216,983,300 | 9,953,164,145 | 97.4 | 9,438,693,084 | 92.4 | 24.8 |
| OfisNP_Capsule_2 | 99.1 |  |  | 99.3 | 11,429,322,000 | 11,131,784,727 | 97.4 | 10,573,587,114 | 92.5 | 25.6 |
