# Supplementary material for: DNA methylation orchestrates secondary metabolite biosynthesis and transport in Papaver somniferum
Source: PLoS One. 2025 Aug 25;20(8):e0329855. doi: 10.1371/journal.pone.0329855 (PMC12377628; doi:10.1371/journal.pone.0329855)
Supplement: S2 Table — (DOCX) [file pone.0329855.s007.docx]

Supplementary Table 2. Alignment efficiency of the samples

| **Samples** | **Raw Reads** | **Clean Reads** | **Alignment efficiency (%)** | **Genome coverage (x)** |
| --- | --- | --- | --- | --- |
| Ofis1_Stem_1 | 38,215,361 | 38,173,057 | 98.0 | 12 |
| Ofis1_Stem_2 | 34,166,560 | 34,148,115 | 92.2 | 12 |
| Ofis1_Stem_3 | 42,254,479 | 42,226,094 | 91.2 | 14 |
| Ofis1_Capsule_1 | 37,624,061 | 37,620,244 | 99.1 | 15 |
| Ofis1_Capsule_2 | 35,078,956 | 35,074,852 | 98.6 | 11 |
| Ofis1_Capsule_3 | 38,295,233 | 38,290,541 | 99.1 | 13 |
| Ofis96_Stem_1 | 41,947,087 | 41,939,986 | 98.9 | 11 |
| Ofis96_Stem_2 | 38,471,381 | 38,467,532 | 99.1 | 11 |
| Ofis96_Stem_3 | 37,453,458 | 37,447,828 | 92.9 | 11 |
| Ofis96_Capsule_1 | 34,702,506 | 34,691,595 | 98.0 | 11 |
| Ofis96_Capsule_2 | 39,449,203 | 39,442,214 | 98.9 | 13 |
| Ofis96_Capsule_3 | 34,326,535 | 34,321,031 | 99.2 | 11 |
| OfisNP_Stem_1 | 34,094,788 | 34,082,122 | 98.4 | 12 |
| OfisNP_Stem_2 | 34,892,821 | 34,866,600 | 96.8 | 16 |
| OfisNP_Stem_3 | 35,526,436 | 35,507,596 | 98.2 | 15 |
| OfisNP_Capsule_1 | 34,056,611 | 34,053,518 | 99.5 | 10 |
| OfisNP_Capsule_2 | 38,097,740 | 38,093,154 | 99.3 | 11 |
